# Melanoma Inhibitory Activity and Melanoma Inhibitory Activity 2 as Novel Immunohistochemical Markers of Oral Epithelial Dysplasia

**DOI:** 10.3390/jcm10163661

**Published:** 2021-08-18

**Authors:** Ryoko Kawai, Yoshihiko Sugita, Toshikatsu Suzumura, Takehiro Hattori, Waka Yoshida, Katsutoshi Kubo, Hatsuhiko Maeda

**Affiliations:** Department of Oral Pathology/Forensic Odontology, School of Dentistry, Aichi Gakuin University, Nagoya 464-8650, Japan; yosshii@dpc.agu.ac.jp (Y.S.); ag203d02@dpc.agu.ac.jp (T.S.); ag193d13@dpc.agu.ac.jp (T.H.); waka@dpc.agu.ac.jp (W.Y.); bobo@dpc.agu.ac.jp (K.K.); hatsu@dpc.agu.ac.jp (H.M.)

**Keywords:** oral epithelial dysplasia, oral potentially malignant disorders, oral squamous cell carcinoma, melanoma inhibitory activity, diagnostic marker

## Abstract

Oral potentially malignant disorders are associated with the development of oral squamous cell carcinoma (OSCC). Most OSCCs are diagnosed via histopathology as oral epithelial dysplasia (OED), but the histologic diagnostic criteria remain non-uniform. Accordingly, the establishment of a diagnostic marker to assist in diagnosis could contribute towards cancer prevention. Melanoma inhibitory activity (MIA) and MIA2 are involved in tumor growth, invasion, and lymph node metastasis in various malignancies. The purpose of this study was to clarify the usefulness of MIA and MIA2 as diagnostic markers of oral mucosal lesions. The expression of MIA and MIA2 was analyzed immunohistochemically in 100 specimens (10 specimens with normal oral mucosa (NOM) and 30 specimens each with low-grade epithelial dysplasia (LED), high-grade epithelial dysplasia (HED), and OSCC). Immunohistochemical results were evaluated based on the Allred scoring system. Cytoplasmic expression of MIA and MIA2 increased in the order of LED, HED, and OSCC. All NOM specimens were negative for cytoplasmic expression. Significant differences were observed between the groups (NOM vs. HED, *p* < 0.05, NOM vs. OSCC, *p* < 0.001). These results demonstrate that MIA and MIA2 are expressed in the oral mucosa within early neoplastic lesions and suggest that MIA and MIA2 are useful novel immunohistochemical markers for discriminating between normal tissue and OED.

## 1. Introduction

The World Health Organization (WHO) classification 2017 describes a new disease concept designated as oral potentially malignant disorders (OPMDs), which are associated with a risk of developing into cancers such as leukoplakia, erythroplakia, or oral lichen planus [1,2,3]. Many OPMDs are pathologically associated with oral epithelial dysplasia (OED), which the WHO classification 2017 describes as a series of structural and cytological changes in the epithelium, caused by the accumulation of genetic mutations that are associated with an increased risk of progression to oral squamous cell carcinoma (OSCC) [2,4]. The risk of malignant transformation of OED increases with increasing grade of OED atypia [5,6]. Accurate pathological diagnosis of OED in OPMDs is therefore important for the prevention of OSCC. The OED histological criteria reported in the WHO classification 2017 are based on architectural and cytological changes [2,3,4]. However, standardized uniform diagnosis of OED is difficult in the absence of a common understanding among diagnosticians. In addition, it is sometimes difficult to determine the transition from normal tissue to OED during routine pathological diagnosis based on the findings of hematoxylin and eosin staining alone. Therefore, novel diagnostic markers of OED are needed in order to ensure accurate diagnosis in routine examinations.

Melanoma inhibitory activity (MIA) is a small secreted protein first identified in malignant melanoma cells and found to promote the transformation of malignant melanoma [7,8]. MIA2 is also a member of the MIA family with homology to MIA [9]. MIA and MIA2 are expressed not only in malignant melanoma but also within various malignant tumors, and their expression appears to increase with malignant transformation [10,11,12]. To date, however, few studies have examined the histopathological differences between normal oral mucosa (NOM), OED, and OSCC in terms of MIA and MIA2 expression.

Identifying changes in MIA and MIA2 expression via the immunohistochemical (IHC) examination of oral lesions related to cancer could contribute to the prevention of oral cancers. An accurate discrimination of borderline lesions between normal tissue and OED is important for selecting appropriate treatments [13]. Therefore, there is an urgent need to identify useful diagnostic markers to assist in the pathological diagnosis of OED. The purpose of this study was to determine whether MIA and MIA2 expression is useful as a diagnostic marker for OED in oral lesions.

## 2. Materials and Methods

### 2.1. Study Population

A total of 100 formalin-fixed, paraffin-embedded (FFPE) biopsy or excised specimens were examined in this study. All specimens were obtained from the Aichi Gakuin University Dental Hospital in Nagoya, Japan. The specimens included 10 NOM, 60 OED, and 30 OSCC samples. In addition, the OED specimens were grouped histologically into low-grade epithelial dysplasia (LED) and high-grade epithelial dysplasia (HED) categories, according to the WHO classification [2]. These were composed of 30 LED and 30 HED specimens. Clinical characteristics of the studied cases are shown in Table 1. All OSCC specimens were diagnosed histologically as grade I according to the WHO classification [1].

### 2.2. IHC Examination

IHC examinations were performed on 4 μm-thick FFPE sections using an anti-MIA polyclonal antibody (Proteintech Japan, Tokyo, Japan) and anti-MIA2 antibody (Abcam, Cambridge, UK). The sections were deparaffinized in xylene, and heat-induced antigen retrieval was performed in Target Retrieval Solution (DAKO Japan, Tokyo, Japan) at 95 °C for 20 min. Endogenous peroxidase activity was quenched by incubating sections in 3% H_2_O_2_ for 10 min. The sections were then incubated with primary antibodies for 60 min at room temperature. Both the anti-MIA and anti-MIA2 antibodies were diluted 1:100. Histofine Simple Stain MAX PO (MULTI) (Nichirei Biosciences, Tokyo, Japan) secondary antibody was used according to the manufacturer’s protocol with similar incubation conditions. Finally, Simple Stain DAB solution (Nichirei Biosciences) was used as the chromogen. Nuclei were counterstained using Meyer’s hematoxylin.

### 2.3. Histopathological and IHC Evaluation

Specimens were considered positive for MIA and MIA2 expression if cells in the tissues exhibited brown staining in the cytoplasm or nucleus. IHC evaluations were performed using antibodies targeting MIA and MIA2 in oral mucosal epithelial cells, following the methodology proposed by Allred et al. [14] and modified in this study. In this study, we assessed positive and negative results using the original scoring criteria shown below. Every IHC-stained specimen was given a proportion score and an intensity score for stained epithelial cells, based on Allred’s score (AS), as follows: AS 0–4, negative; AS 5–8, positive. Intracellular localization of immunohistochemical staining was categorized as cytoplasm only, nucleus only, or both cytoplasm and nucleus.

### 2.4. Statistical Analysis

After the IHC evaluation, differences in staining between specimens were analyzed statistically using the chi-squared test and Fisher’s exact test. We considered a *p*-value of <0.05 to indicate statistical significance.

## 3. Results

### 3.1. IHC Analysis of MIA Expression

Table 2 summarizes the intracellular localization of MIA based on IHC analysis. OED and OSCC specimens exhibited greater staining in the cytoplasm-only category than was observed in the nucleus only category and in the both cytoplasm and nucleus category. OSCC specimens exhibited the highest cytoplasmic positivity (63.3%) among all groups. The expression of MIA in the cytoplasm increased in the order LED, HED, and OSCC. All NOM specimens were negative for cytoplasmic staining. Significant differences were observed between the groups (NOM vs. HED, *p* < 0.05; NOM vs. OSCC, *p* < 0.001; LED vs. OSCC, *p* < 0.01). With regard to nuclear positivity, the expression of MIA was significantly higher in NOM than in LED, HED, and OSCC specimens (NOM vs. LED, *p* < 0.01; NOM vs. HED, *p* < 0.001; NOM vs. OSCC, *p* < 0.001). Positive staining in both the cytoplasm and nucleus was higher in OSCC specimens than in NOM, LED, and HED specimens, but the differences between these groups were not significant.

**Figure 1:**
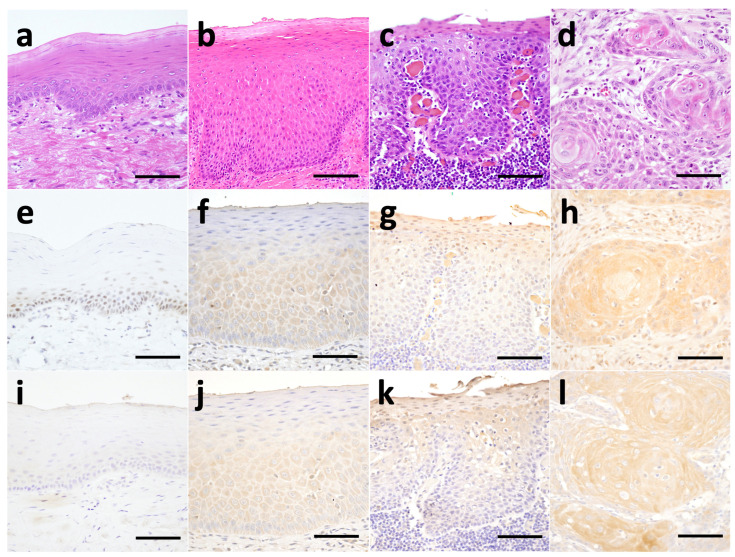
Histopathological features of normal oral mucosa (NOM) (**a**), low-grade epithelial dysplasia (LED) (**b**), high-grade epithelial dysplasia (HED) (**c**), and oral squamous cell carcinoma (OSCC) (**d**). Immunohistochemical staining for MIA and MIA2 in NOM, LED, HED, and OSCC. MIA staining in NOM (**e**), LED (**f**), HED (**g**), and OSCC (**h**). MIA2 staining in NOM (not positive) (**i**), LED (**j**), HED (**k**), and OSCC (**l**). MIA was localized within the nuclei from the basal to lower prickle cell layers in NOM. In LED, HED and OSCC, MIA and MIA2 were mainly localized within the cytoplasm in epithelial cells, LED was observed from the basal to upper prickle cell layers, and OSCC was observed in cell nests. MIA and MIA2 expression were observed up to the epithelial surface in some HED. Scale bar, 100 μm.

No NOM specimens were positive for cytoplasmic MIA staining, but MIA was localized within the nuclei from the basal to lower prickle cell layers (Figure 1e). MIA expression was primarily localized within the cytoplasm from the basal to upper prickle cell layers in OED specimens (Figure 1f). In OED specimens, MIA expression was observed up to the epithelial surface in some cases of HED (Figure 1g). MIA expression was also localized primarily within the cytoplasm in OSCC cell nests (Figure 1h).

### 3.2. IHC Analysis of MIA2 Expression

Table 2 summarizes the intracellular localization of MIA2 based on IHC analysis. OED and OSCC specimens were primarily positive in the cytoplasm. Similar to the expression of MIA, the cytoplasmic expression of MIA2 increased in the order LED, HED, and OSCC. Surprisingly, all NOM specimens were negative for MIA2 expression. Significant differences were observed between the groups (NOM vs. HED, *p* < 0.05, NOM vs. OSCC, *p* < 0.001, LED vs. OSCC, *p* < 0.05). Positive staining in the nucleus was observed only in OSCC specimens. Significant differences were observed between the groups (NOM vs. OSCC, *p* < 0.05; LED vs. OSCC, *p* < 0.001; HED vs. OSCC, *p* < 0.001). Positive staining in both the cytoplasm and nucleus was observed only in OSCC specimens. The differences between the groups were not significant.

No MIA2 expression was observed in the NOM specimens (Figure 1i). MIA2 expression in epithelial cells was localized within the cytoplasm from the basal to upper prickle cell layers in OED cell nests (Figure 1j). In OED specimens, MIA2 expression was observed up to the epithelial surface in some HED cases (Figure 1k). MIA2 expression was also localized within the cytoplasm in OSCC cell nests (Figure 1l).

## 4. Discussion

This is the first study to report the localization of MIA and MIA2 in NOM, LED, HED, and OSCC tissues using immunohistological methods. The most interesting finding of this study was that neither MIA nor MIA2 was expressed in the cytoplasm in NOM specimens. This observation suggests that there are clear differences in MIA and MIA2 expression between NOM and OED tissues resulting from neoplastic changes in OED. This finding suggests that examining MIA and MIA2 expression would be useful for detecting the change from normal tissue to epithelial dysplasia. Previous studies confirmed that MIA is not expressed in benign melanocytes from normal skin biopsies [15,16], but MIA is reportedly expressed in some nevi and all melanoma in situ [15]. These observations suggest that MIA is expressed during the early stages of malignant melanoma development. Once MIA is released from the cells, it binds to fibronectin and integrins, thus directly inhibiting the adhesion of cells to the extracellular matrix. Released MIA then promotes cell migration, tumor invasion, and lymph node metastasis [8,17]. In addition to promoting cell migration, MIA reportedly promotes cell proliferation and inhibits apoptosis [18,19]. Overexpression of MIA enhances tumor cell survival and promotes activation of the PI3K/mTOR signaling pathway. In addition, overexpression of MIA also inhibits the apoptosis of tumor cells [18]. In OSCC, both MIA and MIA2 bind directly to integrins α4β1 and α5β1, and competitively interfere with the binding of integrin ligands. Suppression of apoptosis is mediated by MIA- and MIA2-integrin signaling [20,21].

As a member of the MIA family, MIA2 exhibits amino acid sequence homology to MIA and is specifically expressed in hepatocytes [9]. In the case of hepatocellular carcinoma, MIA2 expression inhibits tumor proliferation [22]. In pancreatic, esophageal, lung, and cervical cancers, however, MIA2 expression is associated with tumor progression [10,23]. Furthermore, when MIA2 is co-expressed with MIA, the overlap of integrin-mediated MIA- and MIA2-associated signaling further promotes tumor progression [20,21].

Members of the MIA family tend to be highly expressed in various types of SCC, such as esophageal, lung, and cervical cancers [10]. MIA and MIA2 are also frequently expressed in OSCC, in which both promote tumor progression [20]. However, their function in precancerous lesions remains poorly understood.

In this study, the expression of MIA and MIA2, particularly in the cytoplasm, was increased in the order LED, HED, and OSCC, but no expression was observed in NOM specimens. These results are consistent with previous studies showing higher expression of MIA and MIA2 in OSCC [19,20], but not in normal epithelial tissues. The expression of MIA and MIA2 expression was strongly upregulated in oral epithelial cells that underwent malignant transformation, including the development of OED. These data suggest that both MIA and MIA2 play an important role in epithelial dysplasia and malignant transformation in the oral mucosal epithelium.

In terms of the pathological features of cytoplasmic expression in oral epithelial cells, MIA and MIA2 expression was observed from the basal to upper prickle cell layers in OED specimens. Some HED cases exhibited expression up to the epithelial surface and in OSCC cell nests. The expression of MIA and MIA2 was only rarely observed in the keratinized layer in OED specimens, but a more pronounced expression was observed in the cancer pearl of OSCC. During the malignant transformation process, the expression of MIA and MIA2 protein may gradually spread into the epithelial cell layer, including the keratinized cells.

In our study, MIA expression was most often localized in the nucleus of NOM specimens. To the best of our knowledge, intracellular localization of the MIA family proteins in oral lesions has not been reported. It is unclear why MIAs localize predominantly in the nucleus of the NOM. Although the proteins may be expressed in the nucleus, they may not be functional in the cells of normal tissues. MIA-positive cells were confined to the basal and parabasal cell layers of the epithelium in NOM specimens. The basal cell layer is characterized by cells that are constantly dividing and proliferating. In addition, the oral mucosa is subjected to chronic stimulation. Some types of stimuli may function as signals for the movement of protein from the cytoplasm to the nucleus in the basal cell layer. In some cases, nuclear expression of MIA was observed in LED, HED, and OSCC specimens. However, only MIA2 was observed in the nucleus of OSCC cells. A correlation between increased expression of MIA and increased expression of the cell proliferation marker proliferating cell nuclear antigen in tumor cells has been reported [18], suggesting that nuclear expression of MIA is related to cell proliferation, but the implications of this correlation are not clear. Further studies on the signaling pathways and signaling genes associated with the MIA family are necessary.

Currently, there is renewed interest in ensuring the correct diagnosis of OED as a means of preventing oral cancer. Diagnosis of OED commonly relies on using a combination of factors, such as CK13, CK17, Ki-67, and p53 [24,25,26,27]. It is widely accepted that CK13 is expressed in normal epithelium but tends to be downregulated by malignant transformation, whereas CK17 is expressed in malignant epithelium. However, these cytokeratin markers are only indicators of epithelial cell differentiation; thus, it is difficult to determine epithelial cell proliferation by examining these markers alone. Generally, OED must be diagnosed by evaluating a combination of cell proliferation markers, such as Ki-67. In contrast, MIA and MIA2, which are not expressed in normal tissues but can be detected in tissues undergoing mild neoplastic changes, appear to be useful novel diagnostic markers for OED. Furthermore, we believe that IHC techniques, which are routine and can be applied immediately in clinical settings, can contribute greatly to the early detection of oral lesions, and thus prevent the development of oral cancers.

In this study, we performed a basic study aimed at confirming the expression of MIA and MIA2 in OED and identified clear differences between NOM and OED. However, there are no reports on these expressions and OED. MIA and MIA2 have been reported to be associated with cell migration, tumor invasion, and lymph node metastasis in OSCC [8,17], however, the role of MIA and MIA2 in OED is the subject of future research. Future studies will need to extend these analyses to clarify the pathophysiology of MIA and MIA2 in OED, which may be useful for the prevention of OSCC. Then, OED patients with MIA and MIA2 expression need to be the follow-up to clarify whether OED patients expressing MIA and MIA2 develop cancer at a higher rate.

## 5. Conclusions

The expression of MIA and MIA2 was observed in the cytoplasm of LED, HED, and OSCC cells, but no cytoplasmic expression was observed in NOM specimens. MIA and MIA2 may therefore be novel IHC markers that enable discrimination between normal tissue and epithelial dysplasia.

## Figures and Tables

**Table 1:** Clinical characteristics of NOM, LED, HED, and OSCC specimens.

Gender	Male (%)		Female (%)	
NOM	4 (40)		6 (60)	
LED	17 (56.7)		13 (43.3)	
HED	17 (56.7)		13 (43.3)	
OSCC	22 (73.3)		8 (26.7)	
**Age (years)**				
NOM	24–77 (Mean ± SD = 52.8 ± 17.3)			
LED	23–85 (Mean ± SD = 61.9 ± 15)			
HED	33–87 (Mean ± SD = 62.9 ± 15.5)			
OSCC	25–83 (Mean ± SD = 58.6 ± 15.5)			
**Site**	**Tongue**	**Gingiva**	**Buccal**	**Palate**
NOM	10	0	0	0
LED	16	5	6	3
HED	24	4	2	0
OSCC	30	0	0	0

NOM, normal oral mucosa; OED, oral epithelial dysplasia; LED, low-grade epithelial dysplasia; HED, high-grade epithelial dysplasia; OSCC, oral squamous cell carcinoma.

**Table 2:** Summary of MIA and MIA2 immunolocalization in NOM, OED, and OSCC specimens.

	NOM *n*(%)	LED *n*(%)	HED *n*(%)	OSCC *n*(%)	*p*-Value
**MIA expression**					
Cytoplasm only	0 (0)	7 (23.3)	11 (36.7) ^a^	19 (63.3) ^ce^	**<0.001**
Nucleus only	7 (70)	4 (13.3) ^b^	3 (10) ^c^	4 (13.3) ^c^	**<0.001**
Both cytoplasm and nucleus	0 (0)	2 (6.7)	2 (6.7)	4 (13.3)	0.681
**MIA2 expression**					
Cytoplasm only	0 (0)	8 (26.7)	12 (40) ^a^	18 (60) ^cd^	**<0.001**
Nucleus only	0 (0)	0 (0)	0 (0)	12 (40) ^afg^	**<0.001**
Both cytoplasm and nucleus	0 (0)	0 (0)	0 (0)	4 (13.3)	**0.039**

NOM, normal oral mucosa; OED, oral epithelial dysplasia; LED, low-grade epithelial dysplasia; HED, high-grade epithelial dysplasia; OSCC, oral squamous cell carcinoma; compared with NOM, ^a^ *p* < 0.05; compared with NOM, ^b^ *p* < 0.01; compared with NOM, ^c^ *p* < 0.001; compared with LED, ^d^ *p* < 0.05; compared with LED, ^e^ *p* < 0.01; compared with LED, ^f^ *p* < 0.001; compared with HED, ^g^ *p* < 0.001; *p*-values were determined using chi-squared test and Fisher’s exact test. Statistically significant differences (*p* < 0.05) are shown in bold font.

## Data Availability

The data obtained in this study are available upon request from the corresponding author. In order to protect the identity of patients, the data are not publicly available.

## References

[1] Reibel J., Gale N., Hille J., Hunt J.L., Lingen M., Muller S., Sloan P., Tilakaratne W.M., Westra W.H., Williams M.D., El-Naggar A.K., Chan J.K.C., Grandis J.R., Takata T., Slootweg P.J. (2017). Oral potentially malignant disorders and oral epithelial dysplasia. WHO Classification of Head and Neck Tumours.

[2] Kujan O., Oliver R.J., Khattab A., Roberts S.A., Thakker N., Sloan P. (2006). Evaluation of a new binary system of grading oral epithelial dysplasia for prediction of malignant transformation. Oral Oncol..

[3] Nankivell P., Williams H., Matthews P., Suortamo S., Snead D., McConkey C., Mehanna H. (2013). The binary oral dysplasia grading system: Validity testing and suggested improvement. Oral Surg. Oral Med. Oral Pathol. Oral Radiol..

[4] Sloan P., Nylander K., Gale N., Reibel J., Hunter K., Salo T., Lingen M., Zain R.B., El-Naggar A.K., Chan J.K.C., Grandis J.R., Takata T., Slootweg P.J. (2017). Tumours of the oral cavity and mobile tongue. WHO Classification of Head and Neck Tumours.

[5] Warnakulasuriya S., Ariyawardana A. (2016). Malignant transformation of oral leukoplakia: A systematic review of observational studies. J. Oral Pathol. Med..

[6] Speight P.M., Khurram S.A., Kujan O. (2018). Oral potentially malignant disorders: Risk of progression to malignancy. Oral Surg. Oral Med. Oral Pathol. Oral Radiol..

[7] Bogdahn U., Apfel R., Hahn M., Gerlach M., Behl C., Hoppe J., Martin R. (1989). Autocrine tumor cell growth-inhibiting activities from human malignant melanoma. Cancer Res..

[8] Bosserhoff A.K. (2005). Melanoma inhibitory activity (MIA): An important molecule in melanoma development and progression. Pigment Cell Res..

[9] Bosserhoff A.K., Moser M., Scholmerich J., Buettner R., Hellerbrand C. (2003). Specific expression and regulation of the new melanoma inhibitory activity-related gene MIA2 in hepatocytes. J. Biol. Chem..

[10] Sasahira T., Kirita T., Nishiguchi Y., Kurihara M., Nakashima C., Bosserhoff A.K., Kuniyasu H. (2016). A comprehensive expression analysis of the MIA gene family in malignancies: MIA gene family members are novel, useful markers of esophageal, lung, and cervical squamous cell carcinoma. Oncotarget.

[11] Bosserhoff A.K., Buettner R. (2002). Expression, function and clinical relevance of MIA (melanoma inhibitory activity). Histol. Histopathol..

[12] Bosserhoff A.K., Hein R., Bogdahn U., Buettner R. (1996). Structure and promoter analysis of the gene encoding the human melanoma-inhibiting protein MIA. J. Biol. Chem..

[13] Messadi D.V. (2013). Diagnostic aids for detection of oral precancerous conditions. Int. J. Oral Sci..

[14] Allred D.C., Harvey J.M., Berardo M., Clark G.M. (1998). Prognostic and predicitive factors in breast cancer by immunohistochemical analysis. Mod. Pathol..

[15] Bosserhoff A.K., Moser M., Hein R., Landthaler M., Buettner R. (1999). In situ expression patterns of melanoma-inhibiting activity (MIA) in melanomas and breast cancers. J. Pathol..

[16] Perez R.P., Zhang P., Bosserhoff A.K., Buettner R., Abu-Ha-did M. (2000). Expression of melanoma inhibitory activity in melanoma and nonmelanoma tissue specimens. Hum. Pathol..

[17] Bosserhoff A.K., Stoll R., Sleeman J.P., Bataille F., Buettner R., Holak T.A. (2003). Active detachment involves inhibition of cell-matrix contacts of malignant melanoma cells by secretion of melanoma inhibitory activity. Lab. Investig..

[18] Gu Q.H., Li D., Xie Z.H., Shen Q.B. (2020). The clinical significance of MIA gene in tumorigenesis of lung cancer. Neoplasma.

[19] Sasahira T., Nishiguchi Y., Fujiwara R., Kurihara M., Kirita T., Bosserhoff A.K., Kuniyasu H. (2016). Storkhead box 2 and melanoma inhibitory activity promote oral squamous cell carcinoma progression. Oncotarget.

[20] Sasahira T., Bosserhoff A.K., Kirita T. (2018). The importance of melanoma inhibitory activity gene family in the tumor progression of oral cancer. Pathol. Int..

[21] Kurihara M., Kirita T., Sasahira T., Ohmori H., Matsushima S., Yamamoto K., Bosserhoff A.K., Kuniyasu H. (2013). Protumoral roles of melanoma inhibitory activity 2 in oral squamous cell carcinoma. Br. J. Cancer.

[22] Hellerbrand C., Amann T., Schlegel J., Wild P., Bataille F., Spruss T., Hartmann A., Bosserhoff A.K. (2008). The novel gene MIA2 acts as a tumour suppressor in hepatocellular carcinoma. Gut.

[23] Kong B., Wu W., Valkovska N., Jäger C., Hong X., Nitsche U., Friess H., Esposito I., Erkan M., Kleeff J. (2015). A common genetic variation of melanoma inhibitory activity-2 labels a subtype of pancreatic adenocarcinoma with high endoplasmic reticulum stress levels. Sci. Rep..

[24] Wils L.J., Poell J.B., Evren I., Koopman M.S., Brouns E.R.E.A., de Visscher J.G.A.M., Brakenhoff R.H., Bloemena E. (2020). Incorporation of differentiated dysplasia improves prediction of oral leukoplakia at increased risk of malignant progression. Mod. Pathol..

[25] Nobusawa A., Sano T., Negishi A., Yokoo S., Oyama T. (2014). Immunohistochemical staining patterns of cytokeratins 13, 14, and 17 in oral epithelial dysplasia including orthokeratotic dysplasia. Pathol. Int..

[26] Ikeda M., Shima K., Kondo T., Semba I. (2020). Atypical immunohistochemical patterns can complement the histopathological diagnosis of oral premalignant lesions. J. Oral Biosci..

[27] Yagyuu T., Obayashi C., Ueyama Y., Takano M., Tanaka Y., Kawaguchi M., Takeda M., Kasai T., Kirita T. (2015). Multivariate analyses of Ki-67, cytokeratin 13 and cytokeratin 17 in diagnosis and prognosis of oral precancerous lesions. J. Oral Pathol. Med..

